# Microbiota–Immune Crosstalk in Pneumonia and Acute Lung Injury: Mechanisms, Evidence, and Therapeutic Opportunities

**DOI:** 10.3390/microorganisms14081758

**Published:** 2026-08-10

**Authors:** Haoran Yuan, Bingyi Li, Caihong Shen, Lixin Xie, Fei Hou

**Affiliations:** 1 The 964th Hospital of PLA Joint Logistic Support Force, Changchun 130062, China; 2 College of Pulmonary & Critical Care Medicine, 8th Medical Center, Chinese PLA General Hospital, Beijing 100091, China; 3 The 83rd Group Army Hospital of the Chinese PLA Ground Force, Henan Medical University, Xinxiang 453003, China

**Keywords:** lung microbiome, gut–lung axis, pneumonia, acute lung injury, acute respiratory distress syndrome, alveolar macrophage, microbial metabolites, probiotics

## Abstract

Mucosal microbiota contribute broadly to host defense and immune homeostasis, while the lung and gut microbiota form a particularly important bidirectional ecological and immunological network that shapes pulmonary host defense, inflammatory injury, and tissue repair. In pneumonia, loss of colonization resistance and altered microbial metabolite production may weaken innate and adaptive immunity; respiratory infection, antibiotics, and critical-care exposures can, in turn, remodel both microbial communities. In acute lung injury (ALI) and acute respiratory distress syndrome (ARDS), intestinal barrier failure, circulating microbial products, immune cell trafficking and, in selected settings, lymphatic or hematogenous dissemination of gut-derived organisms may aggravate alveolar–capillary injury. Alveolar macrophages integrate these signals through pattern-recognition, metabolic, and epigenetic pathways, linking microbial ecology to pathogen clearance and inflammatory resolution. The evidence, however, remains uneven. Mechanistic causality rests largely on animal studies, most human data are associative, and trials of microbiota-directed interventions are heterogeneous and strain-specific. This Review examines bacterial and viral pneumonia, sepsis-associated ALI and ventilator-associated injury; separates mechanistic, observational, and interventional evidence; and evaluates probiotics, live biotherapeutic products, microbial metabolites, and dietary approaches. Translation will depend on longitudinal sampling, source-resolved microbial tracking, metabolite-informed patient stratification, and adequately powered trials with clinically relevant endpoints.

## 1. Introduction

Lower respiratory tract infections remain a major cause of illness and death worldwide, and pneumonia can progress from localized infection to systemic inflammation, acute lung injury (ALI), and acute respiratory distress syndrome (ARDS) [1]. Outcome depends not only on pathogen clearance but also on the host’s capacity to limit collateral inflammation, preserve the alveolar–capillary barrier, and initiate repair. These vulnerabilities arise through distinct mechanisms. In neonates, an immature microbiota and developing immune system limit ecological resilience. In older adults, immunosenescence and inflammaging alter host–microbe interactions. In immunocompromised and critically ill patients, antibiotics, altered nutrition, sedation, aspiration, and mechanical ventilation impose additional immune and iatrogenic disruption. Antimicrobial and supportive therapies remain indispensable, but they do not directly restore the ecological and immune functions disrupted during severe infection. Defining modifiable determinants of immune competence and inflammatory resolution is therefore a central problem in pneumonia and ALI/ARDS research.

Pneumonia was traditionally framed as invasion of a sterile lower respiratory tract by a pathogen. Culture-independent sequencing has displaced that model. The airways instead contain a low-biomass, dynamic microbial community shaped by immigration from the upper airway, local growth conditions, and microbial elimination. Meanwhile, the gastrointestinal microbiota has emerged as a regulator of systemic immunity, hematopoiesis, epithelial integrity, and metabolite availability [2,3,4]. These observations shift the unit of analysis from one pathogen in one organ to an interconnected host–microbial ecosystem [5,6]. Within this ecosystem, disease may reflect not only pathogen burden but also loss of microbial functions, altered host–microbe signaling, and failure to regain ecological resilience after treatment.

The gut–lung axis provides a mechanistic framework for this interdependence. Gut-derived metabolites and microbial products reach the lung through the circulation; intestinal immune education influences the phenotype and recruitment of pulmonary myeloid and lymphoid cells; and severe barrier failure may allow viable enteric organisms to translocate. The traffic is bidirectional. Respiratory infection, systemic inflammation, antibiotics, nutritional change, and mechanical ventilation can each reshape the intestinal microbiota and its metabolic output. In critically ill patients, features of the lung microbial community are associated with ventilator-free days and survival, although these associations do not establish causality [7]. The relevant question is therefore not simply whether the gut and lung communicate, but which route dominates at a given disease stage, which microbial functions are protective or harmful, and which signals can be modified safely.

Interpretation is constrained by several conceptual and methodological problems. Because lower-airway samples contain little microbial biomass, they are highly susceptible to reagent contamination and upper-airway carryover. Detection of DNA does not establish microbial viability, and taxonomic associations cannot distinguish a cause of injury from a consequence of antibiotics, aspiration, ventilation, or organ failure [5]. Even the terms ‘dysbiosis’ and ‘gut–lung axis’ are used inconsistently, at times as descriptive labels rather than testable mechanisms. PMA-assisted sequencing can reduce signals derived from extracellular DNA and membrane-compromised cells, whereas culture, RNA-based metatranscriptomics, and complementary viability assays can provide additional evidence of microbial viability or transcriptional activity [8,9]. However, PMA performance varies with microbial taxa, sample matrix, biomass, and community complexity, and neither PMA-based sequencing nor RNA detection alone provides a definitive quantitative measure of viability in low-biomass respiratory samples [10,11]. Therapeutic claims introduce a second problem: findings from prophylactic animal experiments are often extrapolated to established human disease despite differences in timing, dose, baseline microbiota, immune state, and safety. A clinically useful synthesis must separate mechanistic experiments from observational and interventional evidence and define the boundary between biomarker discovery and therapeutic readiness.

This Review integrates evidence from bacterial and viral pneumonia, sepsis-associated ALI, ARDS, and ventilator-associated injury. We organize gut–lung communication into three routes—soluble microbial products and metabolites, immune cell trafficking, and viable microbial translocation—and consider alveolar macrophages (AMs) as a cellular point of convergence linking microbial ecology to pathogen clearance, inflammatory amplification, efferocytosis, and repair. We then assess probiotics, live biotherapeutic products, purified microbial metabolites, and dietary interventions in relation to evidence level, treatment context, and safety. Distinguishing prevention from treatment, and association from causation, reveals which microbiota-directed strategies have mechanistic support, which are backed by human data, and which remain translational hypotheses.

## 2. The Lung–Gut Microbial Ecosystem

### 2.1. Lung Microbiome

Culture-independent studies have overturned the assumption that healthy lower airways are sterile, revealing instead a dynamic microbial community of low biomass [12,13]. Its composition reflects the balance among microbial immigration, regional growth conditions, and elimination rather than stable local replication. In the adapted island-biogeography model, microaspiration and inhalation introduce microbes, whereas mucociliary clearance, cough, and phagocytosis by AMs remove them [14,15]. Anatomical continuity makes the lower-airway microbiota partly resemble the oral community, while oxygen tension, nutrient availability, pH, airway architecture, and immune tone impose local selection [16,17]. Despite its low biomass, this community may help calibrate respiratory immunity. Commensal-derived signals maintain tonic type I interferon activity and calibrate the activation threshold of innate antiviral responses [18,19,20]. Although the responsible microbial triggers remain incompletely defined, candidate mechanisms include low-level systemic exposure to peptidoglycan fragments, lipopolysaccharides, or microbial nucleic acids, as well as the transport of microbial cargo by bacterial outer membrane vesicles. Direct evidence assigning this basal pulmonary priming to a specific ligand or anatomical route remains limited. Microbial exposure also shapes local T-cell organization and antibody responses [19]. Thus, microbial cues may sustain immune readiness without constitutive inflammation, although the responsible organisms and pathways remain incompletely defined in humans.

The low microbial biomass of lung samples also creates unusual technical vulnerability. Reagent contamination, sampling-device contamination, and upper-airway carryover can each distort community profiles. Bronchoalveolar lavage fluid and whole-lung tissue address different biological questions: in experimental ALI, tissue may improve biomass recovery, whereas lavage more directly samples the airway compartment [21,22]. Negative extraction and procedural controls, quantitative biomass measurements, and paired host response data are therefore essential. Taxonomic shifts alone do not demonstrate viable colonization or causal activity.

Lung dysbiosis has been reported in asthma, chronic obstructive pulmonary disease (COPD), acute respiratory infection, and critical illness [23,24,25,26]. Recurrent features include reduced diversity, expansion of opportunistic taxa, and loss of ecological resilience. In severe bacterial pneumonia and coronavirus disease 2019 (COVID-19)-associated ARDS, enrichment of oral- or gut-associated organisms correlates with persistent inflammation, impaired oxygenation, and poor outcomes [27,28]. These observations are biologically plausible but strongly confounded by antibiotics, aspiration, mechanical ventilation, nutrition, and disease severity. Depending on disease stage and clinical context, dysbiosis may be a cause, a consequence, or simply a biomarker.

Here, the lung microbiome is treated both as a local immunomodulatory ecosystem and as a recipient of systemic microbial perturbation. Three routes connect the gut and lung: circulating metabolites and microbial products, trafficking immune cells and, when barrier integrity fails, translocation of viable organisms. AMs lie at the intersection of these routes and offer a mechanistic link between microbial ecology, pathogen clearance, inflammatory amplification, and repair.

### 2.2. Gut Microbiota

The gut microbiota is the body’s largest and best-characterized microbial ecosystem. In addition to nutrient metabolism and epithelial barrier maintenance, it educates mucosal and systemic immunity through interactions with epithelial cells, dendritic cells, lymphocytes, and resident macrophages [29]. These interactions maintain tolerance to dietary and commensal antigens while preserving rapid antimicrobial responses. Gut-derived signals also regulate systemic immune tone, bone-marrow hematopoiesis, and circulating myeloid cell output. At steady state, tonic, low-level exposure to commensal-derived lipopolysaccharide and peptidoglycan fragments sensed through NOD1 and NOD2 can influence hematopoietic progenitor activity, monocyte output, and myeloid differentiation [30,31,32]. In pneumonia and ALI, the number and phenotype of recruited monocytes and neutrophils can determine whether inflammation clears infection or damages tissue.

Short-chain fatty acids (SCFAs) are prominent systemic mediators of gut–lung communication. Through G protein-coupled receptors and inhibition of histone deacetylases, SCFAs alter myeloid cell development and macrophage gene expression. For example, butyrate can imprint antimicrobial metabolic and transcriptional programs in macrophages [33]. In experimental systems, microbial metabolites induce epigenetic programs consistent with trained immunity and thereby modify later responses to respiratory challenge [34]. Whether analogous programs are stable, beneficial, and therapeutically tractable in patients remains uncertain.

A balanced intestinal ecosystem supports nutrient metabolism, barrier integrity, and immune homeostasis [35]. For example, commensal stimulation of colonic dendritic cells maintains tonic interferon beta (IFN-β) signaling and systemic antiviral resistance [36]. Antibiotics, dietary change, and critical illness disrupt these inputs, but the consequences depend on timing, baseline community structure, and host state [30]. Dysbiosis is therefore better understood as a context-dependent loss of ecological and functional resilience than as a single taxonomic signature.

### 2.3. The Gut–Lung Axis

**Figure 1:**
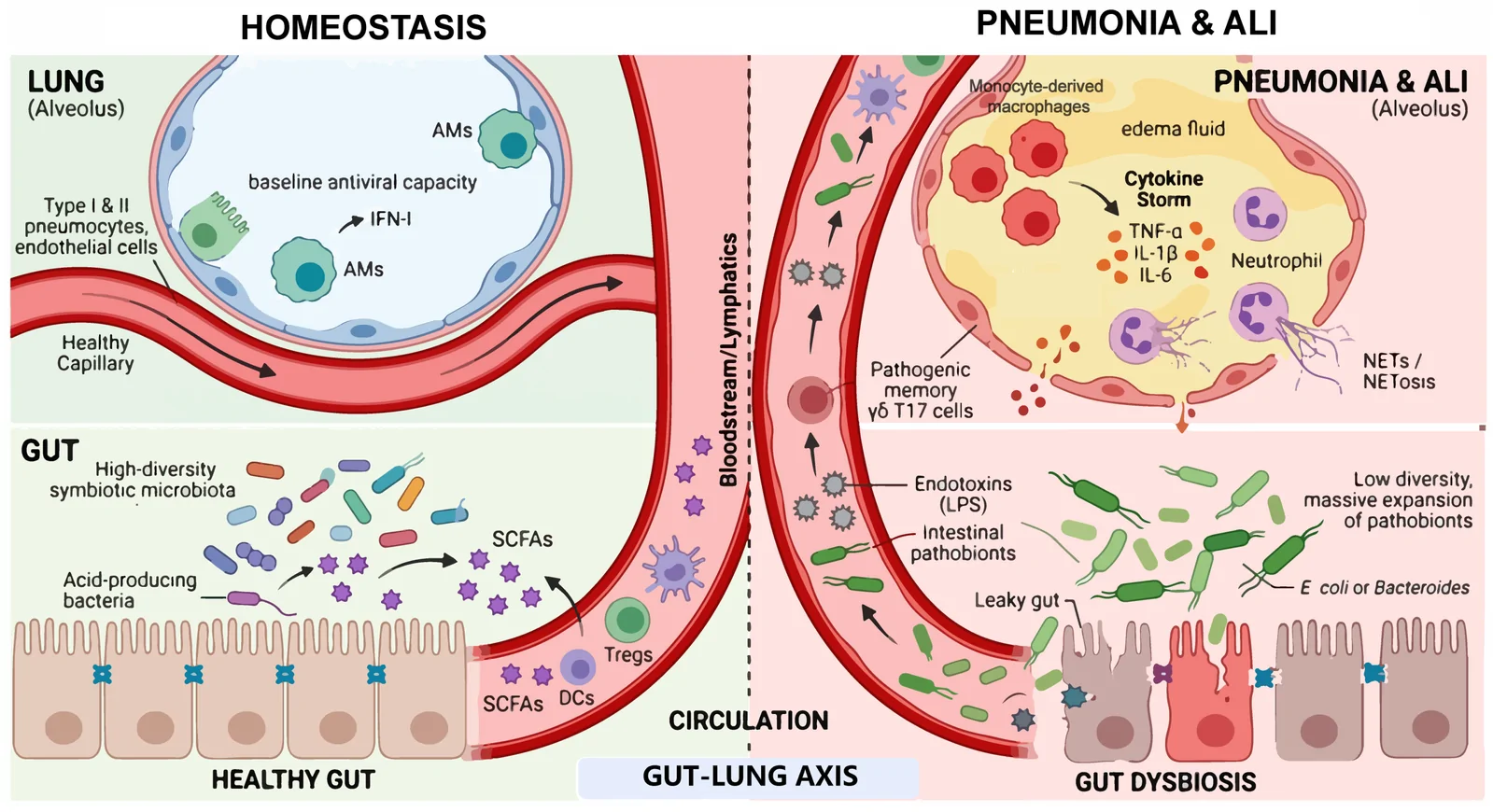
The gut–lung axis in respiratory homeostasis, pneumonia, and acute lung injury. Under homeostatic conditions, a diverse gut microbiota generates metabolites, including short-chain fatty acids (SCFAs), that enter the circulation, tune the functions of AMs, support epithelial barrier integrity, and maintain basal antiviral readiness. During infection, antibiotic exposure or critical illness, dysbiosis and intestinal barrier dysfunction can reduce beneficial metabolites and increase systemic exposure to microbial products. In selected settings, viable enteric organisms and gut-primed immune cells may reach the lung. These inputs may amplify macrophage activation, neutrophil recruitment, NET formation, and alveolar–capillary injury. Solid arrows denote pathways supported by mechanistic experiments; proposed or context-dependent routes require cautious interpretation in humans.

The gut–lung axis denotes bidirectional communication between the gastrointestinal and respiratory tracts through microbial metabolites, immune mediators, neural pathways, and barrier-dependent translocation [37,38]. Gut-to-lung translocation refers to the movement of gut-derived microorganisms or microbial components or products beyond the impaired intestinal barrier, followed by lymphatic or hematogenous dissemination towards the pulmonary compartment. The mesenteric lymphatic route may deliver these signals through the thoracic duct into the systemic circulation, whereas hematogenous dissemination may occur through the portal or systemic circulation, depending on the degree of barrier and hepatic clearance failure. The concept is informative only when directionality and evidence level are preserved: gut-derived signals can alter pulmonary immunity, while lung infection and its treatment can independently reshape the intestinal ecosystem. Under homeostatic conditions, circulating SCFAs and other microbial metabolites tune pulmonary immune responses. During dysbiosis or critical illness, loss of intestinal barrier integrity increases systemic exposure to pathogen-associated molecular patterns such as lipopolysaccharide, which may amplify lung inflammation [37]. This inflammatory amplification may involve neutrophil recruitment and the formation of neutrophil extracellular traps (NETs), which contribute to pathogen containment but can aggravate alveolar–capillary barrier injury when excessive or persistent. Conversely, metabolite and lipid-mediator networks promote macrophage plasticity, efferocytosis, and tissue repair [39,40,41]. Commensals may also strengthen mucosal defense through bacteriocins, antimicrobial peptides, and secretory immunoglobulin A (IgA). The contribution of each route probably differs among pneumonia, sepsis-associated ALI, and ventilator-associated injury (Figure 1).

Studies of asthma and COPD illustrate both the long timescale and the bidirectionality of gut–lung interactions [42]. Early-life depletion of *Lachnospira*, *Veillonella*, *Faecalibacterium*, and *Rothia* has been associated with subsequent asthma risk, whereas antibiotic exposure and low-fiber diets increase allergic airway inflammation in experimental models [3,43,44,45]. The effect of an individual commensal is disease dependent. *Tritrichomonas musculis*, for example, worsens allergic hyperresponsiveness yet improves resistance to pulmonary tuberculosis in mice [46]. Taxa should therefore not be classified as uniformly beneficial or harmful.

Respiratory disease can also disturb the intestine. COPD is associated with reduced gut microbial diversity and impaired intestinal integrity [47]. In mice, respiratory syncytial virus and influenza alter gut community structure [48,49]. Lung-derived type I interferon can deplete obligate anaerobes and favor Proteobacteria, increasing susceptibility to enteric *Salmonella* [50]. Lung-primed C-C chemokine receptor 9 (CCR9)-positive and CD4-positive T-cells can traffic to the small intestine and contribute to interferon gamma (IFN-γ)- and T helper 17 (Th17)-associated injury [51]. These findings establish biological plausibility, but longitudinal human studies are needed to separate the effects of infection from those of drugs, nutrition, and hospitalization.

Host genetic variation is relevant to the interpretation of gut–lung microbiome studies because it can shape baseline microbial community structure, mucosal barrier properties, and immune responsiveness independently of pneumonia or critical illness. It may therefore act as a potential confounder or effect modifier in human observational studies, particularly when cohorts differ in ancestry or population structure. Variants affecting microbial sensing, epithelial glycosylation, and mucosal barrier properties can influence microbial community structure and host responsiveness to microbial signals. In particular, FUT2 and ABO genotypes modify the secretion of mucosal blood-group glycans and are reproducibly associated with the abundance and metabolic capacity of specific gut microorganisms [52], whereas variants in NOD2 may alter microbial recognition and inflammatory responses [53]. Population-based studies have also identified associations involving LCT, HLA-DQB1, and MUC12 [54]. Nevertheless, these findings derive primarily from general population cohorts, and their direct relevance to pneumonia- or ALI/ARDS-associated microbial changes remains uncertain. Future observational studies should therefore consider genetic ancestry and relevant host variants, where feasible, when distinguishing disease-associated dysbiosis from pre-existing host-dependent microbial variation.

## 3. Microbiota in Pneumonia

### 3.1. Dysbiosis Across the Pneumonia Trajectory

Microbial colonization begins after birth, and the lower respiratory tract acquires an early community structure within the first months of life [55]. This period coincides with maturation of local immunity, raising the possibility that disrupted community assembly alters later susceptibility to lower respiratory tract infection. The available evidence is mostly observational, however, and the effects of developmental timing, antibiotics, feeding, and viral encounters are difficult to disentangle.

Pneumonia is accompanied by convergent ecological and functional changes, including reduced colonization resistance, impaired immune priming, and diminished production of immunoregulatory metabolites [56]. Intestinal barrier failure may also permit systemic exposure to microbial products or, in severe illness, translocation of enteric organisms [57]. Community-acquired pneumonia has been associated with reduced alpha diversity and depletion of SCFA-producing taxa, but these findings do not establish causality [57]. In a prospective cohort of 6419 individuals, a greater abundance of butyrate-producing bacteria was associated with a lower incidence of pneumonia [58]. This protective model is testable, but it requires longitudinal validation that accounts for medication exposure.

### 3.2. Bacterial Pneumonia

Depleting gut commensals experimentally weakens early defense against pulmonary bacterial infection. In pneumococcal pneumonia, microbiota-depleted mice develop greater bacterial dissemination, dysregulated cytokine responses, more severe lung injury, and higher mortality; fecal microbiota transfer partially rescues the phenotype [59]. The experiment establishes causality in mice. It does not establish that fecal microbiota transplantation is safe or effective in patients with pneumonia.

The underlying mechanisms are both pathway- and pathogen-specific. During *Klebsiella pneumoniae* infection, antibiotic-induced microbiota depletion impairs early innate responses; systemic nucleotide-binding oligomerization domain-like receptor ligands, but not Toll-like receptor ligands, rescue this defect [60]. Butyrate has also been reported to enhance C-X3-C motif chemokine receptor 1-positive (CX3CR1+) natural killer-cell migration and cytotoxicity through PI3K–AKT signaling, reducing bacterial burden and lung injury in mice [61]. These pathways are candidates for investigation, not clinical interventions ready for use.

Individual commensals also shape pulmonary effector programs. Mice lacking segmented filamentous bacteria are more susceptible to *Staphylococcus aureus* pneumonia, whereas intestinal colonization promotes pulmonary type 17 immunity and bacterial control [62]. Because type 17 responses can also damage tissue, any therapeutic manipulation would need to account for pathogen, timing, and inflammatory state.

### 3.3. Viral Pneumonia

Experimental dysbiosis can compromise antiviral immunity. Streptomycin depletes *Lactobacillus* and *Clostridium* and worsens respiratory syncytial virus-associated lung inflammation, accompanied by altered cytokine production and macrophage polarization [63]. Influenza A virus reduces SCFA availability and increases susceptibility to secondary bacterial infection [64]. Commensal signals also support inflammasome activation, dendritic cell migration and virus-specific T- and B-cell responses [19], whereas the microbial metabolite desaminotyrosine augments type I interferon-mediated protection [65]. Antibiotics may therefore affect both antiviral defense and treatment response, with drug class, exposure window, and baseline microbiota acting as important modifiers [66].

Studies conducted during and after the COVID-19 pandemic established a substantial observational literature linking severe disease to depletion of SCFA-producing commensals and enrichment of opportunistic bacteria or fungi [67,68,69]. Some of this signal may arise from intensive treatment rather than pathogenesis. Dysbiotic signatures should therefore be viewed as candidate prognostic markers, not validated diagnostic tests. In mice, *Akkermansia muciniphila* enhances lung-resident antiviral immunity and inducible bronchus-associated lymphoid tissue during SARS-CoV-2 infection [70]. Controlled human studies are needed before a therapeutic effect can be inferred.

### 3.4. Alveolar Macrophages as Integrators of Microbial Signals

AMs are central to pulmonary surveillance, and systemic microbial cues shape their metabolic fitness and response thresholds. In microbiota-depleted mice, these cells show transcriptional and metabolic abnormalities, respond weakly to bacterial ligands, and phagocytose *Streptococcus pneumoniae* less efficiently [59]. Influenza-associated intestinal dysbiosis and the accompanying loss of SCFAs similarly impair AM antibacterial function, contributing to secondary pneumococcal infection [64].

Microbial signals also influence the transition from pathogen control to inflammatory resolution. Antibiotic-induced dysbiosis favors inflammatory macrophage states during respiratory syncytial virus infection, whereas *Clostridium butyricum* or sodium butyrate partially restores a pro-resolving program [63]. Butyrate likewise limits methicillin-resistant *Staphylococcus aureus*-associated injury in mice, in association with altered macrophage polarization [71]. The conventional classically activated/alternatively activated (M1/M2) labels obscure this complexity; functional, spatial, and single-cell readouts will be more informative.

Inflammatory resolution is active, not a state of passive immunosuppression. Microbial metabolites intersect with host lipid pathways that regulate specialized pro-resolving mediators (SPMs), macrophage efferocytosis, and restoration of the resident macrophage pool [40,72,73]. Direct evidence connecting defined gut organisms to these lipid circuits in human pneumonia remains scarce. At present, the microbiota–lipid connection is a mechanistic frontier rather than an established therapeutic axis.

## 4. Microbiota in Acute Lung Injury and ARDS

ALI and ARDS are defined by inflammatory disruption of the alveolar–capillary barrier. Failure of the intestinal barrier increases circulating lipopolysaccharide (LPS), which activates Toll-like receptor 4 (TLR4) signaling in immune, epithelial, and endothelial cells and amplifies cytokine and chemokine production [74]. Endotoxemia is only one contributor, alongside the initiating insult, endothelial injury, and mechanical stress; it should not be treated as a universal explanation for ARDS.

Gut-derived metabolites can either intensify or restrain lung injury. After intestinal ischemia–reperfusion, succinate may aggravate lung injury through complementary metabolic and receptor-mediated mechanisms. Succinate accumulation during ischemia and its subsequent oxidation by succinate dehydrogenase during reperfusion promote mitochondrial reactive oxygen species production and HIF-1α-dependent inflammatory signaling [75,76]. In addition, microbiota-derived extracellular succinate activates SUCNR1–PI3K–AKT–HIF-1α signaling in AMs, promoting inflammatory activation and secondary pulmonary epithelial apoptosis [77,78]. Autoinducer-2 also aggravates inflammation in mice and correlates with inflammatory markers in patients with pneumonia [79]. By contrast, acetate preserves airway tight junctions through G protein-coupled receptor 43–AMP-activated protein kinase (GPR43–AMPK) signaling during influenza [80], and *Blautia*-derived indole-3-acetic acid protects against sepsis-associated ALI in experimental systems [81]. These opposing effects favor metabolite-resolved analysis over broad claims about a ‘beneficial microbiome’.

Immune cell trafficking and viable bacteria may provide additional routes along the gut–lung axis. In sepsis models, gut-derived interleukin-17-producing gamma delta T (γδT17) cells migrate to the lung and drive interleukin-17A (IL-17A)-dependent injury; AM Wnt–β-catenin signaling and C-C motif chemokine ligand 1 (CCL1) contribute to their recruitment [82]. Bronchoalveolar samples from patients with ARDS and from septic mice can be enriched for gut-associated anaerobes, and their abundance correlates with inflammatory markers [83,84]. Sequence overlap alone is insufficient evidence of translocation; source tracking, viability assays, and contamination controls are also required.

Mechanical ventilation may intensify cross-compartmental disturbance. Whole-genome comparisons in two patients with ventilator-associated pneumonia supported gut-to-lung movement of *Escherichia coli* and *Burkholderia cenocepacia* [85]. The observation is informative but small. In a two-hit model, moderate tidal-volume ventilation after viral injury increased intestinal permeability and activated caspase-11 and NOD-, LRR-, and pyrin domain-containing protein 3 (NLRP3) inflammasome pathways, culminating in gasdermin D-dependent pyroptosis [86]. Subsequent repair depends on coordinated endothelial, epithelial, and macrophage programs, including pro-resolving lipid signaling [87].

## 5. Therapeutic Modulation of the Microbiota

### 5.1. Probiotics and Live Biotherapeutic Products

Microbiota-directed therapies seek to restore ecological function rather than suppress a single inflammatory pathway, but the clinical evidence remains heterogeneous. Trials in preterm infants and healthy adults suggest that selected probiotic or prebiotic preparations reduce some respiratory tract infections or shorten symptom duration [88,89,90,91,92]. Specific regimens have also lowered the reported incidence of ventilator-associated pneumonia in mechanically ventilated patients [93,94]. These effects cannot be generalized across strains, populations, or outcomes, and efficacy and safety in severe COVID-19 and critical illness remain insufficiently established [95].

Probiotic effects depend on strain and context. In mice, probiotic administration enhances AM IFN-β responses during respiratory syncytial virus infection, restores SCFA-producing taxa, and raises circulating SCFA concentrations [96]. *Clostridium butyricum* also shifts cytokine and macrophage responses towards a less injurious state [63]. These observations support biological plausibility, but clinical efficacy cannot be inferred without pharmacokinetic, colonization, and safety data.

Next-generation probiotics and live biotherapeutic products are designed to deliver defined functions, yet evidence in pneumonia remains largely preclinical [97]. *Lactobacillus rhamnosus* modulates neutrophil activation in experimental ARDS [98]. *Microcella aerolata* GA224 protects mice against *Streptococcus pneumoniae* through changes in microbial and metabolic profiles [99], whereas a *Bacillus safensis* metabolite reduces methicillin-resistant *Staphylococcus aureus*-associated injury through Toll-like receptor 2–myeloid differentiation primary response 88–nuclear factor-kappa B (TLR2–MyD88–NF-κB) and nuclear factor erythroid 2-related factor 2 (Nrf2) pathways [100]. In critically ill patients, bloodstream infection, horizontal gene transfer, and unpredictable engraftment are material risks. Product identity and manufacturing quality are therefore central to clinical evaluation.

### 5.2. SCFAs, Other Metabolites, and Dietary Interventions

SCFAs regulate neutrophil, macrophage, and lymphocyte function through receptor-mediated and epigenetic pathways [101]. They inhibit neutrophil extracellular trap formation [102] and enhance macrophage phagocytosis and antimicrobial metabolism [103,104,105]. Supplementation reduces inflammation in several animal models, including age-associated ALI [106], while dietary fiber protects against influenza by altering monocyte hematopoiesis and CD8+ T-cell metabolism [4]. These findings demonstrate target engagement, but the optimal metabolite, dose, route, and treatment window remain undefined.

Other microbial and diet-dependent metabolites expand the range of candidate interventions. Indole-3-acetic acid protects pulmonary microvascular endothelial cells and reduces leakage and inflammation in experimental ALI [107]. The active vitamin D metabolite 1,25-dihydroxyvitamin D3 alters gut community structure and attenuates LPS-induced injury [108]. A ketogenic diet enriches *Lactobacillus* species that convert oleic acid to azelaic acid, promoting AM expansion and neutrophil apoptosis in septic lung injury [109]. Because each intervention has pleiotropic host effects, microbiota dependence should be tested by depletion, transfer, or metabolite-rescue experiments.

**Figure 2:**
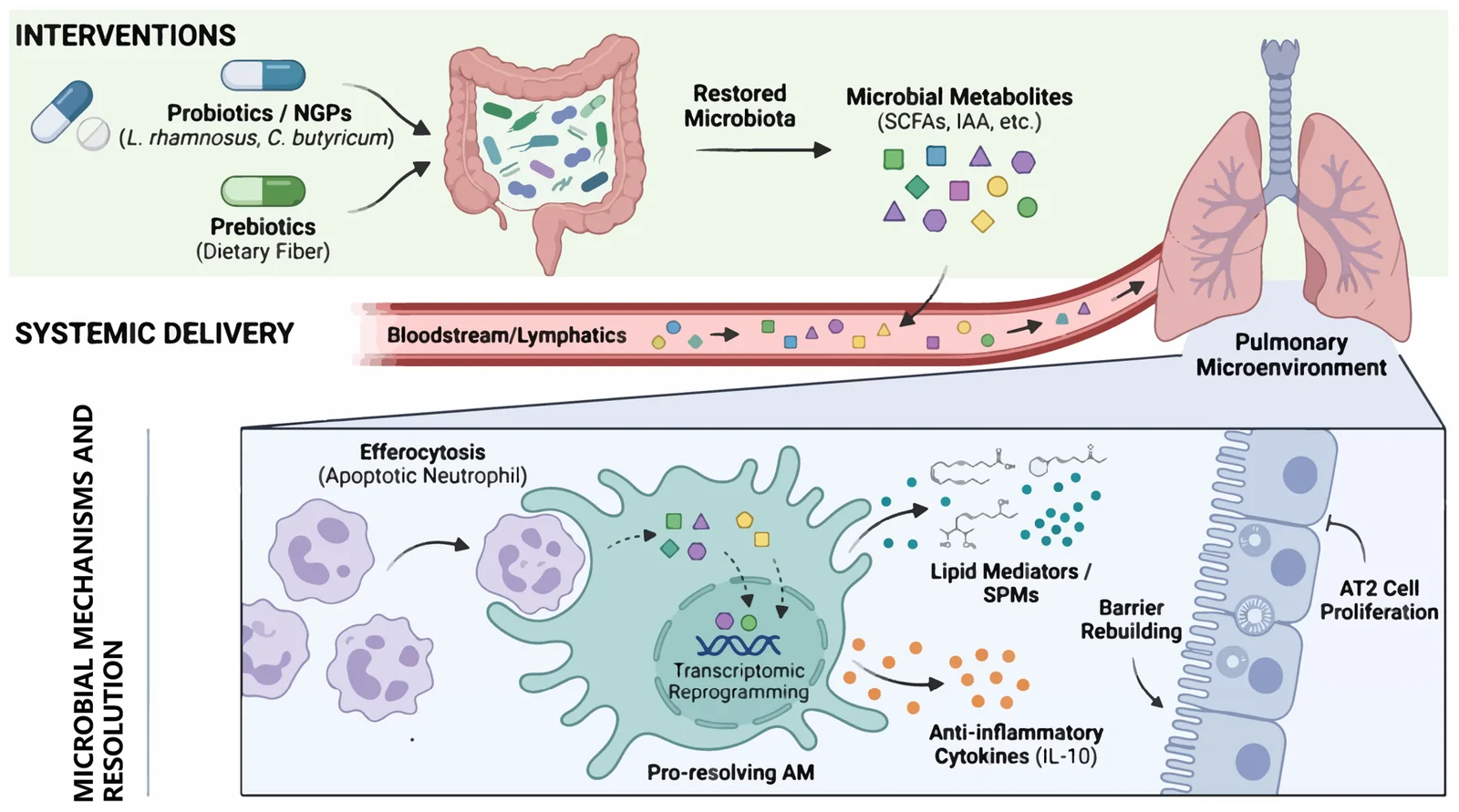
Microbiota-directed strategies and macrophage-centered resolution. Probiotics, live biotherapeutic products, prebiotics, dietary interventions, and purified metabolites may alter intestinal ecology or deliver defined microbial functions. Candidate effectors include short-chain fatty acids (SCFAs), indole derivatives, and lipid-mediator precursors. In experimental models, these signals modify AM metabolism and transcription, promote efferocytosis, restrain neutrophil-mediated injury, and support barrier repair. Evidence strength varies markedly across interventions. Most mechanistic pathways remain preclinical, and live products require dedicated safety assessment in critically ill patients.

Dietary lipid interventions provide another potential route for promoting inflammatory resolution. Omega-3 polyunsaturated fatty acids, particularly eicosapentaenoic acid and docosahexaenoic acid, serve as substrates for the biosynthesis of SPMs [73,110]. Through sequential reactions involving host lipoxygenases, including 5-, 12-, and 15-lipoxygenase, these fatty acids can be converted into resolvins, protectins, and maresins, whereas arachidonic acid gives rise to lipoxins [111,112]. These mediators limit excessive neutrophil recruitment, enhance macrophage efferocytosis, and support tissue repair without broadly suppressing antimicrobial immunity [72,73] (Figure 2). Accordingly, dietary supplementation with polyunsaturated fatty acids may promote resolution by increasing the availability of substrates for pro-resolving lipid-mediator biosynthesis. However, clinical effects are likely to depend on fatty acid composition, dose, timing, host metabolic status, and the enzymatic capacity to generate these mediators.

### 5.3. Translational Evidence and Safety

Across intervention classes, biological plausibility exceeds clinical certainty. Product heterogeneity, limited statistical power, inconsistent endpoints, and incomplete reporting of antibiotic and nutritional exposures hinder pooled interpretation. In critically ill patients, any benefit of ecological restoration must be weighed against infection risk and against the possibility that an intervention effective before infection may be ineffective, or harmful, after organ injury is established (Table 1).

**Table 1:** Translational status of microbiota-directed strategies for pneumonia and acute lung injury.

Strategy	Current Evidence	Potential Clinical Utility/Application	Principal Limitation
Conventional probiotics/prebiotics	Human trials in selected populations [88,89,90,91,92,93,94]	Fewer respiratory infections or ventilator-associated pneumonia (VAP) with selected regimens	Strain-, population-, and endpoint-specific effects; live-organism safety
Defined live biotherapeutics	Mainly animal studies [97,98,99,100]	Immune and metabolic pathway modulation	Engraftment, bloodstream infection, resistance transfer, and manufacturing control
Short-chain fatty acids (SCFAs)	Mechanistic evidence from animal studies [4,102,103,104,105,106]	Macrophage antimicrobial activity, reduced NETosis and barrier support	Dose, route, timing, and off-target effects are unresolved
Other postbiotics	Early-stage preclinical studies [80,81,107]	Defined molecular exposure without live bacteria	Pharmacokinetics, target engagement, and human safety data are sparse
Dietary modulation	Animal and indirect human evidence [4,108,109]	Sustained alteration of metabolite supply	Pleiotropy, adherence, and dependence on baseline microbiota
Microbial diagnostics	Observational cohorts and source-tracking studies [56,57,58,85,113]	Risk stratification and detection of translocation	Confounding, contamination, and lack of prospective clinical utility

## 6. Conclusions and Research Priorities

The gut–lung axis connects microbial ecology with pulmonary host defense, inflammatory injury, and repair. The strongest causal evidence comes from animal studies in which microbiota depletion or defined metabolites alter outcomes in pneumonia and ALI. Human studies add clinically relevant associations among gut taxa, putative translocation signatures, and disease severity, but they have not identified a universal dysbiosis pattern or a microbial target ready for treatment.

Four evidence gaps now define the research agenda. First, longitudinal sampling before and during infection is needed to separate predisposition from treatment-induced dysbiosis. Second, strain-resolved sequencing, culture, or viability assays and source tracking should be integrated with host transcriptomic and metabolomic data. Third, analyses must account for antibiotics, nutrition, ventilation, and other intensive-care exposures. Fourth, mechanistic studies should move beyond coarse taxonomic descriptions and M1/M2 labels towards defined microbial functions, metabolites, and spatially resolved macrophage states. A microbiota–metabolite–lipid–macrophage axis is a plausible working model, but its directionality and therapeutic leverage in humans remain unproven.

Clinical translation should proceed through stratified, mechanism-informed studies rather than empirical microbiota manipulation. Microbial or metabolite signatures may support risk stratification, but prospective validation is required before they can guide diagnosis or treatment [113]. Live biotherapeutic products should be strain defined and evaluated for engraftment, bloodstream infection, and transfer of antimicrobial resistance; purified postbiotics may provide more controllable exposure but still require dose, pharmacokinetic, and safety studies [114]. Informative trials will pair clinically meaningful outcomes with ecological and metabolic measures of target engagement. Such designs can determine whether microbiota modulation complements—rather than replaces—established antimicrobial, ventilatory, and supportive care.

## Data Availability

No new data were created or analyzed in this study. Data sharing is not applicable to this article.

## Abbreviations

ALI, acute lung injury; AMs, alveolar macrophages; AMPK, AMP-activated protein kinase; ARDS, acute respiratory distress syndrome; CCL1, C-C motif chemokine ligand 1; CCR9, C-C chemokine receptor 9; COPD, chronic obstructive pulmonary disease; CX3CR1, C-X3-C motif chemokine receptor 1; GPR43, G protein-coupled receptor 43; HIF-1α, hypoxia-inducible factor 1 alpha; IFN, interferon; IgA, immunoglobulin A; IL, interleukin; LPS, lipopolysaccharide; MyD88, myeloid differentiation primary response 88; NETs, neutrophil extracellular traps; NLRP3, NOD-, LRR- and pyrin domain-containing protein 3; NOD, nucleotide-binding oligomerization domain; Nrf2, nuclear factor erythroid 2-related factor 2; PMA, propidium monoazide; SCFAs, short-chain fatty acids; SPMs, specialized pro-resolving mediators; SUCNR1, succinate receptor 1; Th17, T helper 17; TLR, Toll-like receptor; VAP, ventilator-associated pneumonia.
